# Food-sourcing from on-farm trees mediates positive relationships between tree cover and dietary quality in Malawi

**DOI:** 10.1038/s43016-024-01028-4

**Published:** 2024-08-13

**Authors:** Emilie Vansant, Bowy den Braber, Charlotte Hall, Judith Kamoto, Florian Reiner, Johan Oldekop, Laura Vang Rasmussen

**Affiliations:** 1 Department of Geosciences and Natural Resource Management, University of Copenhagen, Copenhagen, Denmark; 2 Biological and Environmental Sciences, University of Stirling, Stirling, UK; 3 Forestry Department, Lilongwe University of Agriculture and Natural Resources, Lilongwe, Malawi; 4 Global Development Institute, University of Manchester, Manchester, UK

**Keywords:** Geography, Development studies, Agriculture, Forestry, Developing world

## Abstract

Food security policies often overlook the potential of trees to provide micronutrient-rich foods. Here, through causal mediation analysis, we show the positive effect of tree cover on micronutrient adequacy, explained by people sourcing food from on-farm trees. Detailed survey data (*n* = 460 households with repeated surveys) from Malawi were linked to high-resolution (3 m) tree-cover data to capture forest and non-forest trees. Our findings support integrating nutrition and landscape restoration policies.

## Abstract (web summary)

Trees are an important source of food. Combining household surveys with high-resolution land-cover data, a causal mediation analysis shows how sourcing food from on-farm trees mediates the positive effect of tree cover on micronutrient adequacy in Malawi.

## Main

Although global hunger rates have decreased in recent decades, one in three people experience some form of malnutrition, with the highest rates in low- and middle-income countries (LMICs)^1^. Trees are an important source of micronutrient-rich fruits, nuts, seeds and leafy vegetables, and there is increasing recognition of how forests can contribute to dietary quality through the provision of wild foods, income-generating products and ecosystem services that boost agricultural production^2^. However, less is known about the contribution of non-forest trees to improving diets in LMICs, despite the widespread prevalence of trees in agricultural landscapes. The few studies that successfully draw these links are restricted by the use of broad dietary quality indicators (for example, dietary diversity scores, food frequency questionnaires) and therefore do not indicate the degree to which trees, both in forests and outside of forests, support people’s intake of key micronutrients.

**Extended Data Fig. 1:**
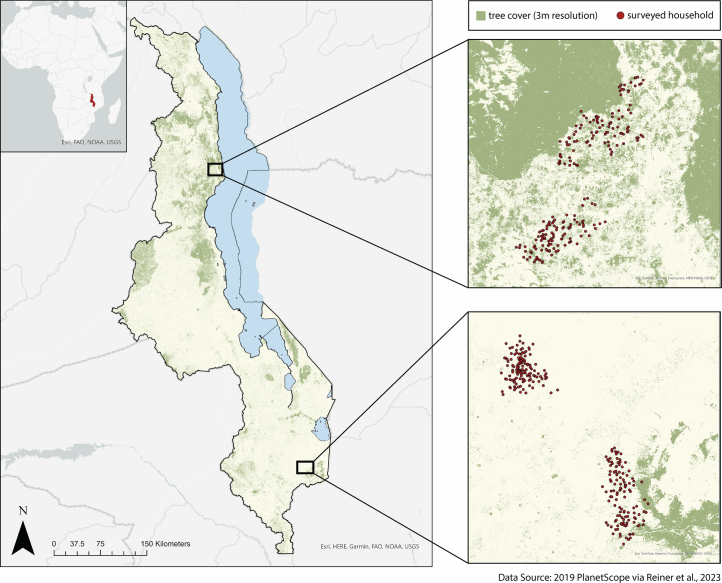
Map of Malawi indicating study sites with surveyed households. Map features surveyed household points overlaid on 2019 PlanetScope data with tree cover predictions at 3 m resolution^4^. Each household is randomly displaced up to 500 m for confidentiality purposes.

In this study, we focus on Malawi, a densely populated country with high rates of poverty and malnutrition, where 51% of the population are classified as severely food insecure and 37% of children under 5 years are stunted^3^. Dependence on subsistence agriculture and wood-sourced fuel has contributed to high deforestation rates in recent decades, with a net loss of 224,000 ha of tree cover (15%) between 2000 and 2022 (ref. ^4^). These combined factors make Malawi an optimal site to study tree–diet linkages. Based on detailed surveys conducted with 460 women in the northern and southern parts of the country (Extended Data Fig. 1), we examine both the effect of overall tree cover and the effect of sourcing food from on-farm trees on women’s micronutrient adequacy (zinc, vitamin A, iron and folate). We conduct a causal mediation analysis to evaluate how the effect of tree cover on diets might be mediated by whether people obtain food from on-farm trees. Causal mediation analysis allows us to measure the extent to which our mediator (sourcing food from trees on farms) transmits the effect of our treatment variable (tree cover) on our dependent variables (zinc, vitamin A, iron and folate adequacy). This approach advances existing knowledge of the positive association between forests and dietary quality^2^ by measuring how much of the tree cover–diet relationship can be explained by the sourcing of food from on-farm trees. Moreover, we advance previous studies by going beyond simple nutrition metrics and measuring women’s micronutrient adequacy.

We base these analyses on an extensive, interdisciplinary dataset combining (1) repeated 24 h dietary recall surveys (on two non-consecutive days within a 7 day period) in both the dry and wet seasons, (2) survey data on households’ decisions to use trees on and around their farmland for food provision, and (3) tree-cover estimates from 2019 PlanetScope imagery (3 m resolution)^5^ within each household’s surroundings.

**Fig. 1:**
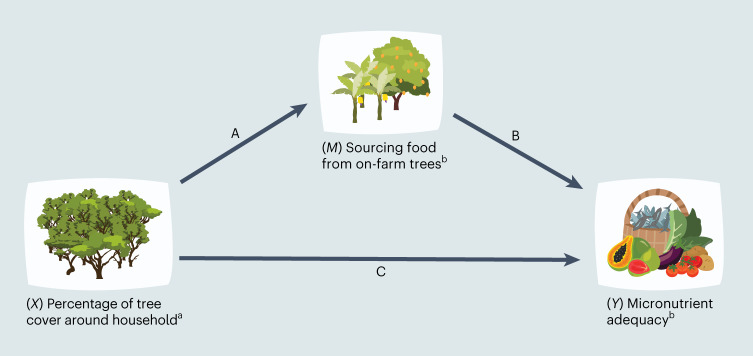
Hypothesized pathways linking tree cover, sourcing food from on-farm trees and people’s dietary quality. The causal mediation analysis^21^ consists of three pathways (corresponding to arrows A, B and C), where the effect sizes for each pathway are used to measure the degree to which sourcing food from on-farm trees (*M*) mediates the relationship between tree cover (*X*) and micronutrient adequacy (*Y*). ^a^2019 PlanetScope data via Reiner et al.^5^. ^b^Household survey data.

Our causal mediation analysis (Fig. 1) reveals four key findings: (1) the amount of tree cover around households is positively associated with women’s micronutrient adequacy levels (path C); (2) greater tree cover also increases the likelihood of whether households source food from their on-farm trees (path A); (3) sourcing food from on-farm trees improves women’s micronutrient adequacy (path B); and (4) the effect of tree cover on women’s adequacy levels for certain micronutrients is partly mediated by use of trees on farms as a food source (paths A + B). We control for the following variables in our models: household size, the Multidimensional Poverty Index (living standards dimension)^6^, farm size (area under cultivation), education level, livestock holdings (tropical livestock units)^7^, crop count and study region. This selection of covariates was informed by a synthesis of studies linking trees and dietary quality^8^ as well as extensive fieldwork in Malawi.

**Fig. 2:**
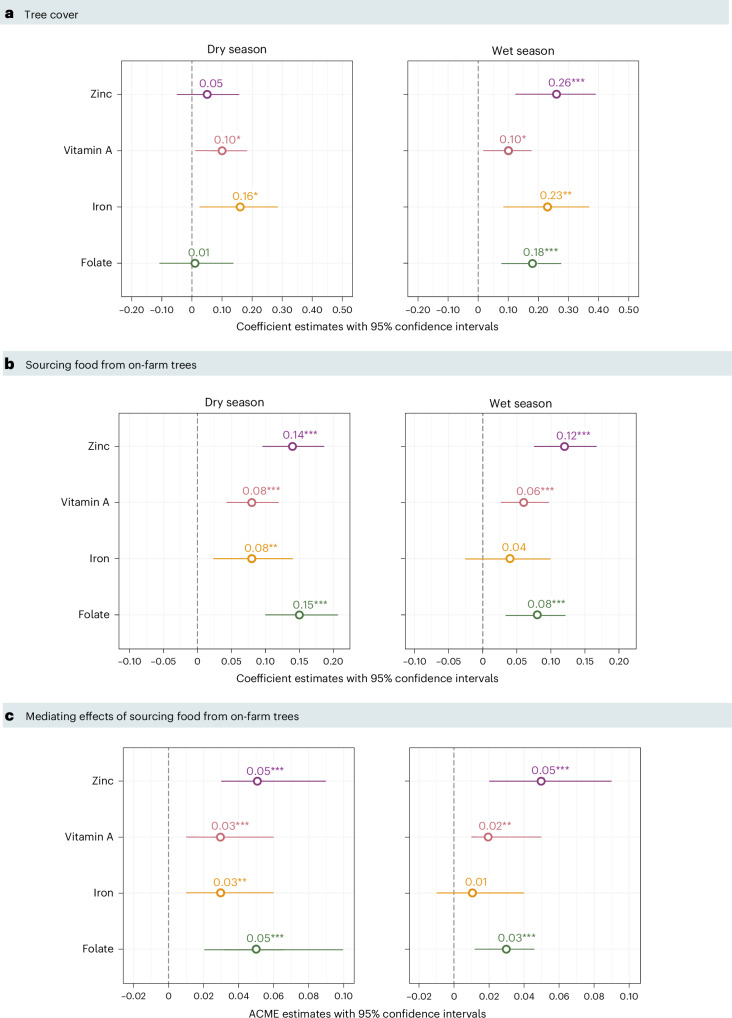
Effect estimates for each component of the causal mediation analysis. **a**–**c**, Results are presented as coefficient effect estimates for linear regression models measuring the effect of tree cover in a 1 km radius around each household on women’s micronutrient adequacy (**a**), logistic regression models measuring the effect of sourcing food from on-farm trees on micronutrient adequacy (**b**) and mediation analysis outputs given as ACMEs, indicating the degree to which sourcing food from on-farm trees mediates the effect of tree cover on micronutrient adequacy (**c**). In all plots, data points are presented as coefficient values within a 95% confidence interval error bar. *P* values are based on two-sided tests. No adjustments were made for multiple comparisons. *n* = 460 women. ***P < 0.001, **P < 0.01, *P < 0.05. Source data

We find positive statistically significant associations between the percentage of tree cover in households’ surroundings and women’s micronutrient adequacy (Fig. 1, path C). For example, we see that a 1% increase in tree cover is associated with a 0.25–0.37% average increase in iron adequacy rates, depending on the season. A jump from 0% to 62% tree cover (maximum observed) corresponds to an increase of 16–23% in overall iron adequacy (dry season, *P* = 0.0192; wet season, *P* = 0.0019) (Fig. 2a). Considering the low mean iron adequacy levels ranging from 64% to 74% (Supplementary Table 1), this indicates how tree cover can contribute to helping women meet WHO recommendations for iron intake (Supplementary Table 2).

When controlling for tree cover, women from households that source food from on-farm trees have higher levels of zinc, vitamin A, iron and folate adequacy in the dry season, and higher levels of zinc, vitamin A and folate adequacy in the wet season compared with women from households that do not source food from on-farm trees (Fig. 1, path B, and Fig. 2b). For example, women sourcing food from trees on their farms have on average 8–15% higher folate adequacy, depending on the season. This suggests that foods from on-farm trees are important sources of folate—especially in the wet season when, on average, folate adequacy is low (48%) (Supplementary Tables 1 and 3).

To better understand whether the effect of tree cover on micronutrient adequacy is mediated by the direct provision of food from on-farm trees, we also test the dependence of the treatment variable (tree cover) on the mediator variable (sourcing food from on-farm trees) (Fig. 1, path A). We find that if tree cover around the household increases by 10%, the probability of using trees on farms for food increases by 9–10% on average. That is, in areas with higher tree cover, households are more likely to source food from on-farm trees.

**Extended Data Fig. 2:**
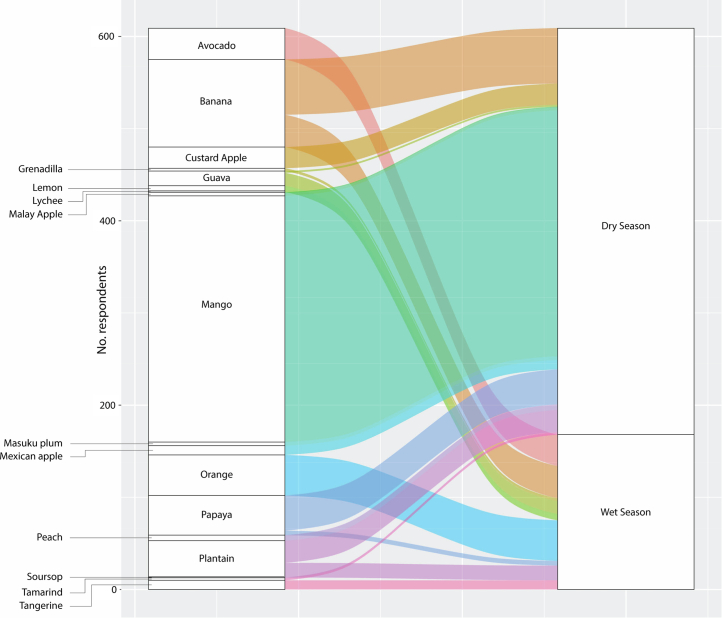
The consumption frequency of different tree-based foods in the dry and wet seasons. The alluvial flow diagram shows the number of women who reported eating different tree-based foods sourced from their own farms in the dry season (n = 441) and wet season (n = 168).

The causal mediation analysis reveals how the direct provision of food from on-farm trees helps to explain the effect of tree cover on micronutrient adequacy. We observe statistically significant average causal mediation effects (ACMEs) for zinc, vitamin A and folate adequacy in both seasons (Fig. 2c and Supplementary Table 4; see Supplementary Table 5 for additional robustness checks). The fact that we do not observe a mediation effect for iron in the wet season—but a positive association between tree cover and iron adequacy—is most likely because areas with high tree cover host wild animals and green leafy vegetables rich in iron, whereas few of the commonly consumed on-farm tree fruits in our study areas have high iron content (for example, bananas, mangoes, oranges, papayas) (Extended Data Fig. 2 and Supplementary Table 3). Yet, high consumption of mangoes, avocado and guava across dry and wet seasons can contribute to improved vitamin A, zinc and folate adequacy (Supplementary Tables 3 and 6).

Ninety-seven percent of Malawians do not have sufficient income to afford a healthy diet on a daily basis^3^. Our results show the nutritional benefits of using trees in agricultural landscapes for direct food provision, thereby identifying an avenue by which households can improve their supply of nutrient-rich foods regardless of income level. However, we note that while trees may produce fruits or nuts annually, monetary and land tenure considerations can influence investment decisions due to the time lag between planting and harvest (Supplementary Information). Access to silvicultural extension services can help farmers reduce this initial investment by enhancing and accelerating tree productivity—which can help ensure the availability of wild and cultivated nutrient-rich foods, especially in areas with limited market access and infrastructure^9^.

Rural Malawians are vulnerable to seasonal fluctuations in food availability^10^. Food shortages in the pre-harvest period, coupled with staple crop price fluctuations, can increase household reliance on self-cultivated or wild foods at certain times of the year. Our results showing how women benefited from different fruits from their on-farm trees in dry and wet seasons (Extended Data Fig. 2 and Supplementary Table 6) can inform reforestation programmes and agroecological interventions^11^. For example, portfolios of socially and ecologically suitable food tree species with staggered harvest periods can be integrated into landscape restoration initiatives (such as AFR100) to leverage the co-benefits of trees for the environment and people’s diets^12^.

Our causal mediation analysis shows how farmers’ decisions to manage trees on their farm for food are influenced by tree cover in the wider landscape—and how trees from forest to farm affect people’s nutrition. These findings lay the groundwork for conservation and restoration policies that address forest and agroforestry systems in tandem. Strengthening the evidence base for tree–diet linkages across more countries and contexts is a key step to aligning food and nutrition initiatives with forest and tree conservation policies and programmes.

## Methods

Malawi is one of the most densely populated countries in Africa, approaching 20 million people with a high annual population growth rate of 3.06%^10^. The majority of the population (83%) lives in rural areas and at least partially depend on smallholder agriculture for their livelihoods. Sixty-two percent of the population was classified as living in multidimensional poverty in 2021^13^, and only 3.7% of people in rural areas have access to electricity^10^. Increasing dependence on natural resources for food, fuelwood and livelihoods has driven widespread deforestation. Reliance on rain-fed food production exacerbates seasonal production variability, leaving farmers highly vulnerable to climatic events (such as droughts and floods) and persistent food insecurity. Consequently, malnutrition rates are high, with widespread deficiencies in zinc, vitamin A, iron and folate, especially among women and children. For example, anaemia prevalence is 31% for women of reproductive age^3^. Taken together, the high levels of natural resource reliance, poverty and malnutrition justify Malawi as a case study country to examine the links between tree cover and dietary quality.

Malawi’s climate is characterized by a unimodal rainy season occurring from December to April. To account for dietary changes based on seasonal food availability, we conducted two rounds of fieldwork in the dry season (October 2021) and wet season (March 2022). Study areas were selected in both a northern region (Nkhata Bay district) and a southern region (Mulanje district) to expand our inferential potential to the country level (Extended Data Fig. 1). The northern study area has a greater percentage of tree cover on customary land, whereas the southern region has a higher population density and less tree cover. In light of recent evidence indicating a relationship between market access and dietary quality in Malawi^14,15^, we selected study sites with relatively equal distance to trading centres of at least 5,000 people.

To measure the extent to which sourcing food from on-farm trees affects people’s dietary quality, we conducted a household survey with 515 initial respondents. Household surveys were administered using Qualtrics XM with questions pertaining to household characteristics, household assets, farming systems, forest use and respondents’ food consumption. We selected women with at least one child between the ages of 2 and 5 years as the primary survey respondents in each household. This was to target women of reproductive age and focus on women feeding their children solid foods. In Malawi, women play an integral role in household food security and nutrition. They are traditionally responsible for food selection, preparation and feeding of dependents (elders/children). Women’s dietary diversity has been shown to align with household dietary diversity^16^, which indicates that women are reliable representatives of dietary quality at the household level. Eligible respondents were selected using systematic sampling (taking every *n*th household on a list, depending on village size). In areas with lower population density, a greater percentage of eligible women were selected to comply with all selection criteria. In especially remote villages, systematic random sampling was not possible due to the limited number of eligible households. In two village areas, all available eligible households were sampled.

We used a quantitative, 24 h dietary recall survey to collect detailed information on the type, quantity and source of the foods people consumed the previous day. For both rounds of fieldwork, each household was visited on two non-consecutive days within a 7 day period. Multiple 24 h recalls at different times of the year have been shown to be useful in accounting for seasonal variation in food intake, and multiple 24 h recalls with the same individual are integral to capturing variability in food intake^17^. To reduce systematic error and bias in the dietary data collection, an interview protocol with culturally sensitive tools and methods was developed in close collaboration with local enumerators. The first visit consisted of conducting a combined household/dietary recall survey, and the second visit consisted of a follow-up dietary recall. For the follow-up dietary recall survey, we obtained attrition rates of 97% (*n* = 499 of 515) and 98% (*n* = 451 of 460) in the dry and wet seasons, respectively. As such, our statistical modelling was based on the 460 respondents for whom we had complete data in both seasons^18^.

Photograph aids and local serving size aids (plates, bowls, cups) were used to help respondents estimate the quantities of food and drink items consumed. Using these same aids, we later converted the local portion sizes into standard units (grams). From the collected food consumption data, we estimated the dietary supply of four key micronutrients: zinc, vitamin A, iron and folate. These micronutrients are of critical nutritional importance and are all targeted by Malawian government policies and interventions due to persistent, widespread deficiencies in the population. Food composition tables for Malawi^19^ were used to estimate the micronutrient content of all food and drink items reported by respondents, and the daily supply of each nutrient was estimated using the consumed quantities in grams. Note that some data were ‘borrowed’ from other food composition tables where data were missing in the Malawian tables (Supplementary Information).

Estimated usual intake values were generated using the multiple source method^20^. This method synthesizes the multiple dietary surveys per respondent to adjust for interpersonal variation in consumption patterns. To estimate the adequacy of each micronutrient to meet minimum requirements, we used recommended nutrient intake (RNI) values^21^. RNI values were estimated based on each respondent’s age and pregnancy and breastfeeding status in both the dry and wet season surveys (Supplementary Table 2). We then compared the RNI against each respondent’s estimated usual intake (intake/RNI, capped at 1) to calculate the nutritional adequacy ratio for each micronutrient. To assess the mean adequacy level for all micronutrients of interest, we also calculated the mean adequacy ratio for each respondent and for our study population overall in each season (Supplementary Table 1).

We use tree cover in the landscape (including trees inside and outside forests) as a treatment variable. Tree cover was calculated as a percentage within a 1 km radius around each household location, aggregated from a very high resolution map of African tree cover in 2019^4^. The continental tree-cover map was created using a deep learning model to segment tree cover at the individual tree level, based on 3 m resolution PlanetScope satellite imagery. The use of very high resolution imagery notably allows the mapping of individual non-forest tree crowns, such that both forest trees and trees outside forests are included in the tree cover. Note that while tree cover was used as a treatment variable for testing paths C and A, we also controlled for tree cover (as a covariate) for testing path B (Fig. 1).

We focused on the use of trees on farms for food as a binary mediator. In the dry season household survey, respondents were asked if they had trees on or around their farmland (‘around’ was defined as within 15 m of the field boundary). If they responded ‘yes’, they were asked if their household sources food from these trees for household consumption (not to be conflated with using food from trees on farms for commercial purposes or owning food tree seedlings from which they cannot yet harvest food). This variable therefore centres on the households’ decision to use trees on and around their farmland for household food provision. As the question on tree use did not specify use during a specific time period, data on how households use on-farm trees were only collected in the dry season, with the assumption that presence/use of on-farm trees is not dependent on seasonality.

It is also acknowledged that women’s access to resources is influenced by land tenure systems. In Malawi, land inheritance is patrilineal in the northern region and matrilineal in the southern region (Supplementary Information). Although different land tenure systems must be considered in any intervention, our analysis operates on the assumption that the participants, by saying they are using the trees on their farm for food, had access to those trees.

**Extended Data Fig. 3:**
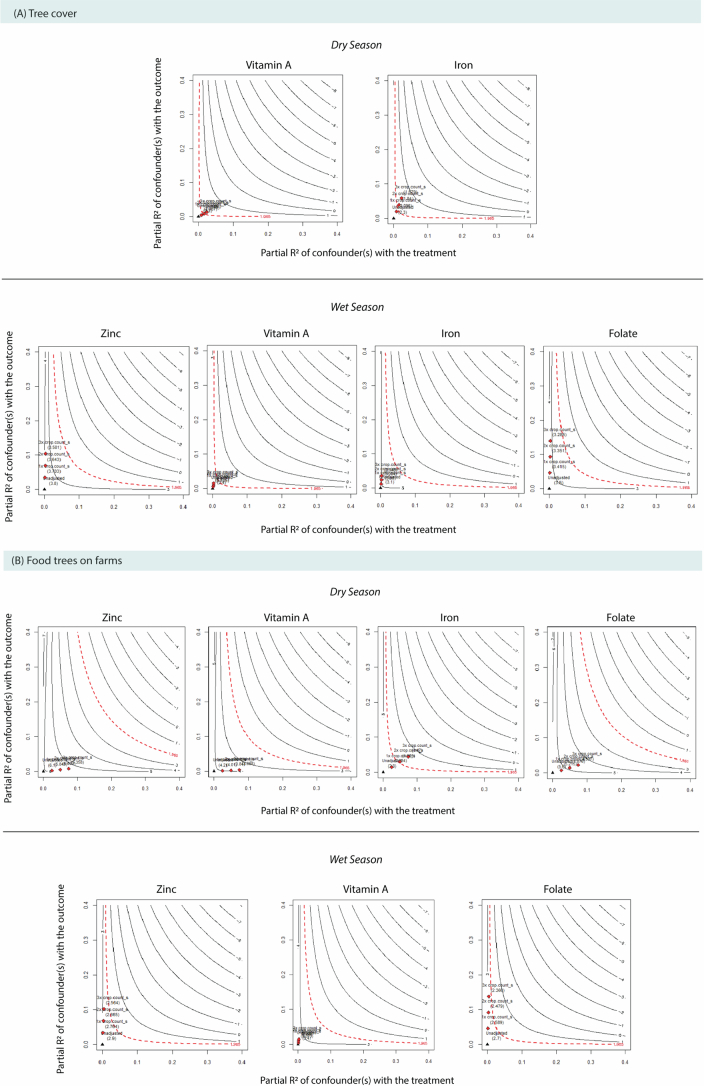
Sensitivity contour plots of t-values to test for omitted variable bias. Sensitivity tests using tree cover (A) and sourcing food from on-farm trees (B) as treatments to model effects on micronutrient adequacy levels (n = 460 women).

We used the ‘mediation’ package in R to evaluate the average causal mediation (that is, indirect) effect of our food tree variable^22^. We tested the significance of the indirect effects using bootstrapping procedures, where unstandardized indirect effects were computed for 1,000 bootstrapped samples with 95% confidence intervals. We then conducted sensitivity analyses to test for omitted variable bias (Extended Data Fig. 3), the robustness of the ACME estimates (strong confounding effects between the mediator and outcome) (Supplementary Table 7), and the consistency of model results using a 500 m tree-cover buffer radius around each household (Supplementary Table 4). As an additional robustness measure, we conducted the causal mediation analysis using the mean adequacy ratio as an outcome (Supplementary Table 5) to check for consistency in model trends.

**Extended Data Fig. 4:**
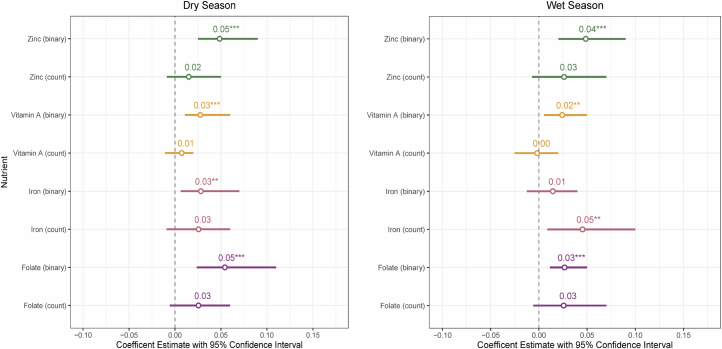
Comparison of Average Causal Mediation Effects (ACMEs) when using ‘sourcing food from on-farm trees’ and ‘number of food tree species’ as mediator variables. Coefficient plots comparing ACME estimates for how a) the degree to which sourcing food trees on farms (binary mediator) and b) the unique food tree species count per household (count mediator) mediates the relationship between tree cover and micronutrient adequacy. Note: the analysis using the unique food tree species count mediator was conducted only with women from households that source food from on-farm trees (n= 360 women). In all plots, data points are presented as coefficient values within a 95% confidence interval error bar. P values are based on two-sided tests. No adjustments were made for multiple comparisons. See Supplementary Table 8 for exact P values. ***P<0.001; **P<0.01.

In the dry season household survey, respondents also reported the different food tree species they currently cultivated. To explore if the diversity of on-farm food tree species could explain the relationship between tree cover and micronutrient adequacy, we conducted a supplementary analysis equivalent to the second step of a hurdle model. Here we only selected households that source food from their on-farm trees (*n* = 360) to avoid conflation with food trees used only for income purposes, and we excluded households that did not source food from on-farm trees (*n* = 100). We then conducted a causal mediation analysis with this subsample, using food tree species count as a mediator. The results (Extended Data Fig. 4) show that higher food tree diversity has a statistically significant positive effect on iron adequacy in the wet season (*P* = 0.0090), but not for any of the other micronutrients (Supplementary Table 8). This indicates that the decision to source any food from on-farm trees has a larger effect on micronutrient adequacy than the number of food tree species.

### Reporting summary

Further information on research design is available in the Nature Portfolio Reporting Summary linked to this article.

### Supplementary information

Supplementary Information Supplementary Tables 1–8, Methods, Context and References.

Reporting Summary

### Source data

Source Data Fig. 2 Statistical source data.

## Data Availability

The dataset generated by the survey research for the replication of this study is available in a Harvard Dataverse repository and can be accessed at 10.7910/DVN/WBUTCK (ref. ^18^). We also accessed very high-resolution (3 m) tree-cover data from 2019 PlanetScope nanosatellite constellation imagery^4^. Estimated nutrient intakes were calculated using the following publicly available food composition tables: Malawi, http://hdl.handle.net/10427/D217R336D; Tanzania, https://nutritionsource.hsph.harvard.edu/food-tables/; Zambia, https://nfnc.org.zm/download/zambia-food-composition-tables-4th-edition/; Mozambique, http://hdl.handle.net/10138/337295; Kenya, https://nutritionhealth.or.ke/programmes/healthy-diets-physical/food-composition-tables/; West Africa, https://openknowledge.fao.org/server/api/core/bitstreams/c5b37ac2-7082-48ab-a4a5-68d27deb4849/content; United States (USDA), https://fdc.nal.usda.gov/. Source data are provided with this paper.

## Code availability

The code written to complete the analyses and ensure the replication of this study is available in a Harvard Dataverse repository and can be accessed at 10.7910/DVN/WBUTCK (ref. ^18^).
