# Supplementary material for: Food-sourcing from on-farm trees mediates positive relationships between tree cover and dietary quality in Malawi
Source: Nat Food. 2024 Aug 13;5(8):661–6. doi: 10.1038/s43016-024-01028-4 (PMC11343702; doi:10.1038/s43016-024-01028-4)
Supplement: Supplementary file 1 — Supplementary Tables 1–8, Methods, Context and References. [file 43016_2024_1028_MOESM1_ESM.pdf]

nature food

Supplementary information https://doi.org/10.1038/s43016-024-01028-4

Food-sourcing from on-farm trees mediates positive relationships between tree cover and dietary quality in Malawi

In the format provided by the authors and unedited

Food-sourcing from on-farm trees mediates positive relationships between tree cover and dietary quality in Malawi

Vansant, Emilie[1], den Braber, Bowy[1]; Hall, Charlotte[1,2], Kamoto, Judith[3], Reiner, Florian[1], Oldekop, Johan[4], Rasmussen, Laura Vang[1]

[1] Department of Geosciences and Natural Resource Management, University of Copenhagen, 1350 K, Denmark
[2] Biological and Environmental Sciences, University of Stirling, Stirling, U.K.
[3] Forestry Department, Lilongwe University of Agriculture and Natural Resources, Lilongwe, Malawi
[4] Global Development Institute, University of Manchester, Manchester, UK

Corresponding author: Emilie Vansant, ecv@ign.ku.dk

Supplementary Information

1. **Supplementary Tables**
2. **Supplementary Methods**
3. **Supplementary Context**
4. **References**

1. Supplementary Tables

Supplementary Table 1. Summary statistics for control and outcome variables. Values are presented as the mean value with the standard deviation in parentheses unless otherwise noted. Temporally independent variables (collected at one point in time) are merged across seasons. Note: tree cover is measured as the percentage of tree cover within a 1km radius around each household.

	Dry Season	Wet Season
Total respondents [n]	460	460
Region [% of households, n]	North (46.1, 212); South (53.9, 248)	
Tree cover [% within 1 km buffer]	0.18 (0.15)	
Use of on-farm trees for food [% of households, n]	Yes (78.3, 360); No (21.7, 100)	
MPI living standards [range 0-1]	0.57 (0.2)	
Education level [% of households, n]	None (2.6, 12); Primary (74.4, 342); Secondary (23, 106)	
Household size	5.84 (2.65)	
Farm size (acres)	1.37 (1.1)	1.08 (0.88)
Crop count	4.05 (1.81)	4.52 (1.92)
Livestock (TLU)	0.19 (0.25)	0.11 (0.22)
Zinc adequacy	0.85 (0.19)	0.70 (0.26)
Vitamin A adequacy	0.93 (0.16)	0.93 (0.15)
Iron adequacy	0.74 (0.24)	0.64 (0.27)
Folate adequacy	0.64 (0.24)	0.47 (0.19)
Mean Adequacy Ratio (MAR)	0.79 (0.15)	0.69 (0.16)

Supplementary Table 2. Recommended nutrient intakes (RNIs) and recommend safe intakes (RSIs) for survey respondents based on age, pregnancy, and breastfeeding status[1]. The lowest bioavailability for zinc (15%) and the mid-level bioavailability level for iron (10%) were used based on firsthand observations of typical diet composition in the study areas, reflecting a mix of animal and plant derived foods.

	Zinc (RNI) mg/day	Vitamin A (RSI) µg RE/day	Iron (RNI)* mg/day	Folate (RNI) µg/day
Age Group: 18+	9.8[2]	500[3]	29.4	400
Pregnant	15[4]	800	29.4[6]	500
Breastfeeding	16.97[5]	850	15	600

[1]Data sourced from: World Health Organization & FAO. *Vitamin and Mineral Requirements in Human Nutrition*. (2004).
[2] For adolescents 18 years of age, the RNI is 14.4 mg/day
[3] For adolescents 18 years of age, the RSI is 600 µg RE/day
[4] An average of zinc RNIs for 1st, 2nd, and 3rd trimesters of pregnancy
[5] An average of zinc RNIs for 0-3, 3-6, and 6-12 month phases of lactation
[6] No figures are given for dietary iron requirements in pregnant women because the iron balance in pregnancy depends not only on the properties of the diet but also and especially on the amounts of stored iron.

Supplementary Table 3: Micronutrient content of most common tree-based fruits grown and consumed by respondents.

Fruit	Source FCT	Zinc (mg)	Vitamin A (mcg)	Iron (mg)	Folate (mcg)	Average fruit size (g)[2]
Mango (ripe)	Malawi	0.54	123.00	1.70	34.00	211
Banana	Malawi	0.20	4.00	0.30	20.00	70
Avocado	Malawi	0.30	6.00	0.70	33.00	270
Custard Apple	Malawi	1.40	2.00	0.70	0.00	225
Guava	Malawi	0.20	5.00	0.40	19.00	108
Orange	Malawi	0.22	8.00	0.30	30.00	151
Papaya	Malawi	1.29	16.00	3.90	15.00	998
Plantain	Malawi	0.10	38.00	0.80	13.00	78

[1]Data sourced from: Malawian Food Composition Table[12]
[2]Average fruit weight after removal of peel and seed, sourced from local markets in study areas

Supplementary Table 4: Coefficient table for robustness check using 500m radius tree cover buffer compared to 1km tree cover buffer. The results are presented as coefficient effect estimates derived from linear regression models examining the effect of tree cover (corresponding to the models featured in Figure 2A) and logistic regression models examining the effect of sourcing food from on-farm trees (corresponding to the models featured in Figure 2B) on women's adequacy levels of different micronutrients. Results compare model outputs using a 500m vs. 1km tree cover buffer variable. P values are based on two-sided tests. No adjustments were made for multiple comparisons.
P values: '***' < 0.001; '**' < 0.01; '*' < 0.05, '+' < 0.1

Treatment	Buffer	Dry				Wet			
		Zinc	Vitamin A	Iron	Folate	Zinc	Vitamin A	Iron	Folate
Tree cover (Fig. 2A)	500m	0.02 (0.7620)	0.10 (0. 0367) *	0.13 (0.0917) +	0.04 (0. 5469)	0.21 (0.0083) **	0.06 (0. 2280)	0.22 (0.0087) **	0.16 (0.0058) **
	1km	0.05 (0.31315)	0.10 (0.0272) *	0.16 (0.01923) *	0.01 (0.8146)	0.26 (0.0002) ***	0.10 (0. 0177) *	0.23 (0.0019) **	0.18 (0.0005) ***
Sourcing food from on-farm trees (Fig. 2B)	500m	0.14 (<0.0001) ***	0.08 (<0.0001) ***	0.09 (0.0038) **	0.15 (<0.0001) ***	0.13 (<0.0001) ***	0.07 (0.0003) ***	0.04 (0. 2010)	0.08 (0.0003) ***
	1km	0.1 (<0.0001) ***	0.08 (<0.0001) ***	0.08 (0.0061) **	0.15 (<0.0001) ***	0.12 (<0.0001) ***	0.06 (0.0007) ***	0.04 (0. 2539)	0.08 (0.0005) ***

Supplementary Table 5. Coefficient table for robustness check using Mean Adequacy Ratio (MAR) as an outcome in a causal mediation analysis. The results are presented as coefficient effect estimates derived from linear regression (Treatment: Tree cover), logistic regression (Treatment: Sourcing food from on-farm trees), and causal mediation analysis (ACME estimates). P values are based on two-sided tests. No adjustments were made for multiple comparisons.
P values: '***' < 0.001; '**' < 0.01; '*' < 0.05, '+' < 0.1

Treatment	Dry	Wet
	MAR	MAR
Tree cover (Fig. 2A)	0.08 (0.0502) +	0.19 (<0.0001) ***
Sourcing food from on-farm trees (Fig. 2B)	0.11 (<0.0001) ***	0.07(<0.0001) ***
ACME estimates (Fig. 2C)	0.04 (<0.0001) ***	0.03 (<0.0001) ***

Supplementary Table 6: The effects of owning common fruit tree species on women's micronutrient adequacy. The results are presented as coefficient effect estimates derived from logistic regression. P values are based on two-sided tests. No adjustments were made for multiple comparisons.
P values: '***' < 0.001; '**' < 0.01; '*' < 0.05, '+' < 0.1

MANGO (n = 300)	Vitamin A	Zinc	Iron	Folate
Dry	0.04 (0. 0097)**	0.07 (0. 0002)***	0.05 (0. 0644) +	0.11 (<0.0001)***
Wet	0.04 (0. 0097)**	0.07 (0. 0047)**	0.01 (0. 6934)	0.05 (0. 0137)*
BANANA (n = 193)				
Dry	0.00 (0. 9684)	0.04 (0. 0325)*	0.03 (0. 1927)	0.04 (0. 0743) +
Wet	0.02 (0. 1669)	0.04 (0. 1253)	0.02 (0. 5135)	0.02 (0. 2554)
PAPAYA (n = 139)				
Dry	0.00 (0. 8331)	0.04 (0. 0313)*	0.01 (0. 7326)	0.03 (0. 2128)
Wet	0.01 (0.4300)	0.03 (0. 2491)	0.03 (0. 3291)	0.01 (0. 4196)
AVOCADO (n = 143)				
Dry	0.03 (0. 0425)*	0.03 (0. 0967) +	0.02 (0. 4076)	0.04 (0. 1377)
Wet	0.00 (0. 9270)	0.05 (00558) +	0.04 (0. 1075)	0.03 (0. 0786) +
GUAVA (n = 80)				
Dry	0.00 (0. 9450)	0.04 (0. 0855) +	0.03 (0.2700)	0.07 (0. 0133)*
Wet	0.02 (0. 2888)	0.09 (0. 0035)**	0.07 (0. 0281)*	0.07 (0. 0034)**

Supplementary Table 7. Direct mediation effects of sourcing food from on-farm trees. The values of the sensitivity measure ρ are shown at which the estimated average causal mediation effects (ACMEs) are zero. P values are based on two-sided tests. No adjustments were made for multiple comparisons.
P values: '***' < 0.001; '**' < 0.01; '*' < 0.05, '+' < 0.1 ; ADE = Average Direct Effect

	Dry Season				Wet Season			
	Zinc	Vitamin A	Iron	Folate	Zinc	Vitamin A	Iron	Folate
ADE	-0.01 (0.8100)	0.06 (0.1500)	0.12 (0.1120)	-0.06 (0.3500)	0.20 (0.0140) **	0.07 (0.0160) *	0.21 (0.0060) **	0.14 (0.0080) **
Total Effect	0.05 (0.4700)	0.09 (0.0240) *	0.15 (0.0360) *	0.00 (0.9700)	0.24 (<0.0001) ***	0.09 (<0.0001) ***	0.22 (0.0020) **	0.16 (<0.0001) ***
ACME(ρ)	0.5	0.3	0.2	0.5	0.3	0.3	0.1	0.3

Supplementary Table 8. ACME estimates for how a) the degree to which sourcing food trees on farms (binary mediator, n = 460 women) and b) the unique food tree species count per household (count mediator, n = 360 women) mediates the relationship between tree cover and micronutrient adequacy. Note: the analysis using the unique food tree species count mediator was conducted only with women from households that source food from on-farm trees. P values are based on two-sided tests. No adjustments were made for multiple comparisons.
P values: '***' < 0.001; '**' < 0.01; '*' < 0.05, '+' < 0.1

Type of Mediator Variable	Dry				Wet			
	Zinc	Vitamin A	Iron	Folate	Zinc	Vitamin A	Iron	Folate
Binary (n = 460 women)	0.05 (<0.0001) ***	0.03 (<0.0001) ***	0.03 (0.0060) **	0.05 (<0.0001) ***	0.04 (<0.0001) ***	0.02 (0.0040) **	0.01 (0.2640)	0.03 (<0.0001) ***
Count (n = 360 women)	0.01 (0.250)	0.01 (0.4100)	0.03 (0.1100)	0.03 (0.1020)	0.03 (0.1060)	0.00 (0.8500)	0.05 (0.0090) **	0.03 (0.1340)

2. Supplementary Methods

Ethics and Inclusion

This study was conducted as part of the FORESTDIET project, funded by the European Research Council (ERC) under the European Union's Horizon 2020 research and innovation programme (Grant agreement No. 853222). The project has received approval from the ERC and the Research Ethics Committee for Science and Health at the University of Copenhagen (hosting institution).

In line with the Global Code of Conduct for Research in Resource-Poor Settings, this study was designed, executed, and reported in collaboration with local researchers at the Lilongwe University of Agriculture and Natural Resources (LUANAR) in Malawi. Prior to the commencement of the study, a formal collaboration was established with Dr. Judith Kamoto (co-author), Vice Principal of Bunda College at LUANAR. Fieldwork was conducted in collaboration with six MSc Nutrition students at LUANAR, providing a capacity-building opportunity for local researchers skilled in dietary data collection. Partnerships were also established with local District Forest Offices (DFOs) in each study district prior to data collection. Consent forms in the local language were read and signed by survey participants to ensure voluntary participation and optional withdrawal at any time in the study prior to data collection.

Study site selection

Study areas in Malawi were selected in both a northern region (Nkhata Bay district) and a southern region (Mulanje district) for expanded inferential potential. The northern region has a greater percentage of tree cover on customary land, whereas the southern region has a higher population density and less tree cover[2] (Supplementary Figure 1). Within these regions, we used the Global Forest Watch platform to identify areas in each region that had experienced similar degrees of tree cover change from 2000 – 2021[3]. To ensure a degree of consistency in access to intact forests, in each region we selected a study area adjacent to a forest reserve: The Kaning'ina forest reserve (Nkhata Bay) and the Mulanje Mountain forest reserve (Mulanje) are both composed of predominantly indigenous miombo woodland. Kaning'ina covers an area of about 143 km^2 (71 km^2 of forest cover) and has a mean elevation of 1134m above sea level. Mulanje covers an area of 585km^2 (184 km^2 of forest cover) with a mean elevation of 1574m above sea level [4]. Both reserves are governed under the National Forest Policy, which permits the collection of non-timber forest products (such as foods and medicinal plants) by communities [5].

Village areas were then selected within these regions based on forest reserve and market proximity. To assess how proximity to an intact forest may influence food consumption, households in each region were surveyed in two sites comprised of village clusters. These sites were located at two different distances to either the Kaning'ina or Mulanje forest reserves, at 0-2 km or 5-6 km from each reserve. This latter threshold of proximity was considered outside the usual 'resource shed' of forest areas, determined by the average distance traveled by forest-dependent communities to collect forest products [6,7]. Considering recent evidence indicating a relationship between market access and dietary quality in Malawi [8,9], we selected sites with relatively equal distance to trading centers of at least 5,000 people [10]. Each site had mobile food and textile markets within 1 km from the village areas (usually within the village center) and a permanent trading center within 5 km. Finally, the village areas selected in each study region were ethnically homogenous, with the village area populations predominantly (>90%) identifying as either Tonga/Tumbuka (Nkhata Bay) or Lomwe (Mulanje). It is noted that in terms of food culture, Tonga and Tumbuka cultures are regarded as similar based on mutual linguistic intelligibility and shared cultural history relative to other ethnic groups in Malawi [11].

Dietary data collection and processing

The household and 24-hr dietary recall surveys were tested and validated by a team of six MSc Nutrition graduates from the Lilongwe University of Agriculture and Natural Resources (LUANAR), who were hired as enumerators to conduct the surveys in the local language, Chichewa. All enumerators also contributed to the translation and revision of the household and dietary data survey tools.

We equipped each enumerator with several tools to aid the respondent in recalling exactly what and how much they ate the day before. Prior to starting the survey in each study area, a visual (photo) aid of common local foods was compiled in order to help respondents correctly recall portion sizes of single food items, such as maize porridge

or cooked cassava. All fruits/vegetables were weighed both with and without inedible portions (i.e. peels/seeds), and the latter was used in estimating consumption in grams. We also used local serving size aids (bowls, cups, plates) to help respondents estimate the amounts consumed. Serving size aids were presented during the survey and given to the respondent to help them show the portion size of different food items consumed (e.g. 0.5 medium bowl, 0.75 small cup, etc.). These tools were also later used to help convert portion sizes of different foods into grams.

Post-data collection, the raw survey data was anonymized and aggregated to generate a unique food item list. The amount of each food item per unit (in grams) was estimated based on photo aid measurements, weighed cooked recipes, recipe documentations, proxies from similar foods/recipes of similar density, and food composition databases. Each unique food item (both single and composite) was matched with a corresponding item from published food composition tables (FCTs) and their corresponding nutrient profiles. The Malawi FCT[12] was used as the primary reference, which is one of the most recent and comprehensive National Food Composition databases of LMICs. Missing values/items not found were sourced from the following FCTs: Tanzania[13], Zambia[14], Mozambique[15], Kenya[16], West Africa[17], and United States (USDA)[18]. Using these values, we calculated the nutrient intake for each food item reported as consumed.

We statistically converted our intake records for each respondent into estimated usual intakes using the Multiple Source Method (MSM) web program[19]. The MSM method estimates usual dietary intakes for individuals using repeat dietary recalls to adjust for day-to-day within-person and between-person variation in consumption patterns[20]. Such an adjustment can better capture episodically consumed foods and nutrients, thereby reducing systematic and random measurement errors in 24-hr recall protocols with limited repeat measurements per respondent[21].

Covariates

We selected covariates with confounding potential based on a review of relevant literature[22] as well as fieldwork observations in order to maximize the potential of our models to make causal inferences. In terms of potential confounding variables, previous studies have found relationships between dietary quality and poverty level[23], education[24,25], and household size[26,27]. We used the Multidimensional Poverty Index (MPI) living standard dimension, which varies from 0 to 1, with 1 being most deprived[28]. We calculated the index according to six indicators: 1) assets (TV, telephone, computer, animal cart, bicycle, motorbike, refrigerator, car/truck), 2) electricity, 3) sanitation, 4) cooking fuel, 5) water source, and 6) housing. Highest level of education achieved was recorded for each respondent, with a range of 'none', 'primary', 'secondary' and 'diploma'. We also included three agricultural variables (crop species count, livestock holdings, farm size) given the evidence supporting positive associations between agricultural diversity and dietary quality[29–33] and the influence of farm area on dietary diversity[34]. Crop species count and livestock holdings were based on self-reported assessments by each respondent relative to the last growing season. We then calculated the amount of livestock owned per household in tropical livestock units (TLU), a standardized indicator of livestock production and productivity[35]. Finally, farm size was measured as the respondents' self-reported number of acres cultivated in the last growing season as an indicator of agricultural production capacity. We also controlled for the study region due to the differences in population density between the two study districts[2].

Causal Mediation Analysis

We conducted a causal mediation analysis[36] to estimate how tree cover in proximity to the household (X) is associated with micronutrient adequacy (Y), and to what degree this association is mediated by food trees on farms (i.e. the direct provision pathway) (M) (Fig. 1). This required running three linear regression models to test 1) the direct effect of tree cover (X) on the decision to source food from on-farm trees (M); 2) the direct effect of tree cover (X) on micronutrient adequacy (Y); and 3) the direct effect of sourcing food from on-farm trees (M) on micronutrient adequacy (Y) while controlling for tree cover (X). In all three models, we controlled for study region, living standard (MPI), education level, household size, farm size (no. acres cultivated), crop count, and livestock holdings (TLUs). For steps 2 and 3, we ran eight separate models in each step because we a) have four outcome variables of interest: zinc adequacy, vitamin A adequacy, iron adequacy, and folate adequacy, and b) distinguish between the dry season and the wet season. The '*mediation*' package in R allows us to estimate the proportion of the direct effect between our treatment and outcome that is attributable to the mediator[37]. However, simulation studies[38,39] have shown relatively weak estimates of significance (and unusually wide confidence intervals) for proportion mediated values for sample sizes < 500, and therefore this metric was not included in our results.

Sensitivity analyses

To confirm the robustness of our ACME estimates, we test for a possible confounding effect of unmeasured pre-treatment variables. Here, a large sensitivity parameter (ρ) indicates a potential violation of the key sequential ignorability assumption underlying the mediation analysis via the existence of strong confounding effects between the mediator and outcome[40] (Supplementary Table 7). Our treatment variable, tree cover, was derived from 2019 satellite data, and thus temporally precedes our mediator. The sensitivity parameters for our ACME estimates reflect a 'modest' degree of sensitivity to pre-treatment confounding across outcomes and seasons (Supplementary Table 7). Given the observational nature of this study, our ACME estimates remain robust to the effect of confounders and do not represent a significant violation of the sequential ignorability assumption. We also conduct a sensitivity analysis to test for the impact of omitted variables for the models in Fig. 2A and Fig. 2B. We tested for the impact of omitted variables using the 'sensmakr' package developed by Cinelli and Hazlett (2020)[41]. We generated sensitivity contour plots of t-values (Supplementary Figure 3) to illustrate the effect of unobserved confounders on our results based on our observed benchmark covariate: crop count (selected as a benchmark as it most frequently emerged as significant in the model results). We found our effect estimates remain robust to unobserved confounding, even with the addition of confounders at strengths exponentially higher than the crop count covariate. Finally, we conducted a robustness check by replacing our treatment variable (1km tree cover buffer) with a 500m tree cover buffer in our input models, finding results to be consistent with those from our models using the 1km forest cover buffer (Supplementary Table 4).

Understanding the role of food tree species diversity

In light of existing research supporting links between agricultural diversity and nutrition[32], we assessed whether diversity of food tree species affected women's micronutrient adequacy (measured by the unique count of food tree species grown by each household). To do this, we conducted a hurdle model, where we selected all households that source food from their on-farm trees (to avoid conflation with food trees used only for income purposes) (n = 360). We then conducted the causal mediation analysis with this subsample, using unique food tree species count as a mediator (Supplementary Figure 4). As the only significant result was the effect on iron adequacy in the wet season, the decision to source food from on-farm trees (the mediator for the main analysis) appears a greater determinant of micronutrient adequacy than food tree species diversity.

3. Supplementary Context

Malnutrition in Malawi

The country faces significant challenges in terms of food security and nutrition, with widespread micronutrient deficiencies among women and children (see Online Methods). An analysis of the 2016/2017 national Fourth Integrated Household Survey (IHS4) found low consumption of nutrient-rich foods (based on a 7-day food consumption survey): less than 40% of households regularly consumed any meat, eggs, or dairy products, while fruit was reported to be consumed by only about half of all households [42]. Ninety-four percent of Malawi's majority rural population relies on smallholder agriculture for livelihoods [43]. Consequently, most Malawians' food consumption is shaped by trends in smallholder food crop production [10]. As such, policies and practices concerning the intersecting fields of agriculture and national resource management are some of the complex contributors to high malnutrition rates in Malawi.

Agricultural practices

Due to lack of alternative livelihood options in rural areas, over 80% of the working population is engaged in rain-fed, smallholder agriculture, leading to high dependence on natural resources for sustaining livelihoods[44]. Smallholders produce 80% of domestically consumed food, with the principal crops being maize, rice, cassava, legumes, and potatoes (sweet and Irish)[45]. Maize occupies half of all cultivated land countrywide and is grown by more than 95% of farming households[46]. Yet continuous cropping of maize, low use of improved agricultural inputs, and increasing population pressure has led to steady declines in soil fertility[47]. As a result, almost 40% of agricultural land in Malawi is degraded[48].

While many farmers retain scattered trees on and around their farmland, adoption of agroforestry systems remains limited. A study on agroforestry adoption in Malawi finds that while the use of fertilizer trees on farms improved

farmers' food security, the decision to adopt fertilizer trees on farms was largely determined by farmers' access to resources and information – including education on the long-term benefits of tree adoption to soil fertility[47]. With regard to fruit tree species, consultation with agroforestry experts at LUANAR revealed that barriers to fruit tree adoption stem principally from institutional bottlenecks (e.g. limited road networks, availability of quality seedlings from nurseries) rather than farmers' lack of technical knowledge. For example, seedlings of common fruit tree species, such as mango, are sold to farmers after experts/extension workers have grafted them at the nursery. Fruit tree seedlings are therefore usually provided to the farmers post-grafting, with simple instructions on planting, spacing, and watering. Farmers are advised to plant the trees at the beginning of the rainy season to ensure the trees do not require extra irrigation while in the seedling stage.

The cost of purchasing and maintaining fruit tree seedlings depend on the vendor and species in question. For example, grafted mango tree seedlings sell on average for 1,000 – 1,500 MWK ($0.6 – 0.9 USD). Hybrid mango seedlings that can produce large mangos with high market value are generally more expensive (up to 5,000 MWK) while local mango seedlings are usually cheaper (500 MWK) and are therefore perceived as more financially accessible. Maintenance expenses are a bit more difficult to estimate, especially as the "invisible" labor costs are context-dependent. According to our local collaborators, most tree species are self-managed and require only occasional weeding around the base. Pesticide use on trees is generally limited to aphid infestations, which are very rare.

To help address institutional barriers to agroforestry adoption, NGOs and government extension agencies can promote silvicultural training and tree-planting schemes at farm and landscape levels to ensure increased availability of wild and cultivated nutrient-rich foods, especially in areas with limited or imperfect market access. In Malawi, breeding programs for wild fruit trees have resulted in accelerated fruit production, substantially reducing the time lag between planting and harvest of indigenous fruits such as baobab (*Adansonia digitata*), safou (*Dacryodes edulis*), and wild loquat (*Uapaca kirkiana*)[49]. Such innovations, when coupled with extension and technical support, can encourage farmer adoption and investment in on-farm trees for food production.

Land Tenure

Local-level variations in household land tenure security can hinder households from investing in tree planting. In Malawi, land is inherited through customary tenure systems, defined as either patrilineal (through the male line) - dominant in the northern region - or matrilineal (female line) - dominant in the southern region. In both arrangements, the death of a landowner can trigger processes of expropriation by which certain household members lose access to land[50]. Measures to 'informally formalize' customary land rights through policy reformations neglect inequities in land access and perpetuate power structures in which the local authorities can make decisions regarding land inheritance - decisions that can be corrupted by local politics [51]. These formalization processes follow trends of land commoditization. In Malawi, there has been an increase in the amount of rented land, where parcels are loaned usually for just one cropping season due to the fear that any longer would give the renter claim over the field[51]. These informal, lineage-based, and gender-biased practices contribute to land tenure insecurity and discourage land investments that can deliver long-term benefits for the environment and agricultural productivity, such as planting trees.

In our study population however, only 19% of surveyed households rented land that they cultivated – with most of these households cultivating on a mix of owned and rented land. From these households, on average only 12% of their cultivated land was rented, with households reporting that they often rented land for cultivation to supplement and extend their farm area. Indeed, only 20 households (4%) cultivated exclusively on rented land. Interestingly, 16 out of the 20 households that cultivated exclusively on rented land also sourced food from on-farm trees. This indicates that land tenure may not be an obstacle to accessing food from on-farm trees.

Food culture

In Malawi, maize (particularly in porridge form) is treated as a dietary requirement, where other nutrient-dense foods (fruits, vegetables, legumes) are regarded as more 'optional' accompaniments[52]. This common perception of tree-based foods as "supplementary" to maize may inform farmers' decisions to cultivate on-farm trees. Indeed in some cases, the cultivation and consumption of nutritious foods is restricted by traditional beliefs: such as taboos against growing and consuming bambara nuts[53], or eating eggs while pregnant[54]. Despite Malawi hosting a diversity of ethnicities, such social practices that might have originated from one culture have become widespread in Malawian society, partially due to increases in inter-regional migration[55]. Such belief systems may also dictate

whether crops are consumed or sold. For example, interventions to improve diet diversity have found that increased food availability of nutritious crops (e.g., groundnuts, soya beans) did not always translate into nutrition gains for families, as these crops were often sold and the revenue used for maize or non-food purchases[52,56]. In times of low-agricultural production (i.e. the lean season), households may prioritize purchasing more 'durable' staple foods that can quickly meet basic energy needs, such as maize flour, bread buns, or biscuits. Wild foods (e.g. fruits, insects, and green leafy vegetables) are also commonly consumed during the lean season. Despite evidence that forests support wild food consumption in Malawi[57], our study participants conveyed that wild foods are often stigmatized as "poor people's food." In this way, dietary preferences and cultural norms intersect with economic considerations to perpetuate dietary homogeneity.

4. References

1. Reiner, F. et al. More than one quarter of Africa's tree cover is found outside areas previously classified as forest. Nat. Commun. 14, 2258 (2023).

2. National Statistical Office. 2018 Malawi Population and Housing Census Main Report. (2019).

3. Global Forest Watch. Malawi Deforestation Rates & Statistics. https://www.globalforestwatch.org/dashboards/country/MWI (2023).

4. European Commission. DOPA Explorer - Malawi. Digital Observatory for Protected Areas (DOPA) Explorer https://dopa-explorer.jrc.ec.europa.eu/ (2023).

5. Government of Malawi. National Forest Policy. https://www.dof.gov.mw/storage/app/media/Policies%20and%20Strategies/National%20Forest%20Policy%202016.pdf (2016).

6. Kefa, C. A., Gregory, A., Espira, A. & Lung, M. Does Wood Fuel Gathering for Household Use Follow an Optimality Model? A Study from Kakamega Forest, Western Kenya. Hum. Ecol. 46, 473–484 (2018).

7. Maukonen, P., Donn, P. & Snook, L. K. Addressing Potential Conflict Using Participatory Mapping: Collection of Forest Foods From Timber Trees Around Industrial Concessions in Cameroon. Front. For. Glob. Change 3, (2020).

8. Koppmair, S., Kassie, M. & Qaim, M. Farm production, market access and dietary diversity in Malawi. Public Health Nutr. 20, 325–335 (2017).

9. Matita, M. et al. Does household participation in food markets increase dietary diversity? Evidence from rural Malawi. Glob. Food Secur. 28, 100486 (2021).

10. IFPRI. Agriculture, Food Security, and Nutrition in Malawi: Leveraging the Links. https://ebrary.ifpri.org/digital/collection/p15738coll2/id/132311 (2018).

11. Kamwendo, G. Language, Identity and the Politics of Recognition in Post-Banda Northern Malawi. Ufahamu J. Afr. Stud. 31, (2005).

12. MAFOODS. Malawian Food Composition Table 2019. (Lilongwe, Malawi, 2019).

13. Lukmanji, Z. et al. Tanzania Food Composition Tables. (2008).

14. Republic of Zambia. Zambia Food Composition Tables: 4th Edition. (2009).

15. Korkalo, L., Hauta-alus, H. & Mutanen, M. Food composition tables for Mozambique. (2011).

16. FAO/Government of Kenya. Kenya Food Compostion Tables. (2018).

17. FAO. FAO/INFOODS Food Composition Table for Western Africa. (2020).

18. USDA. FoodData Central. U.S. Department of Agriculture, Agricultural Research Service https://fdc.nal.usda.gov/ (2019).

19. Harttig, U., Haubrock, J., Knüppel, S., Boeing, H., & EFCOVAL Consortium. The MSM program: web-based statistics package for estimating usual dietary intake using the Multiple Source Method. Eur. J. Clin. Nutr. 65 Suppl 1, S87-91 (2011).

20. Tooze, J. A. Estimating Usual Intakes from Dietary Surveys: Methodologic Challenges, Analysis Approaches, and Recommendations for Low- and Middle-Income Countries. (2020).

21. Gibson, R. S., Charrondiere, U. R. & Bell, W. Measurement Errors in Dietary Assessment Using Self-Reported 24-Hour Recalls in Low-Income Countries and Strategies for Their Prevention. Adv. Nutr. Bethesda Md 8, 980–991 (2017).

22. Vansant, E. C. et al. What are the links between tree-based farming and dietary quality for rural households? A review of emerging evidence in low- and middle-income countries. People Nat. 4, 296–311 (2022).

23. Ickowitz, A., Powell, B., Salim, M. A. & Sunderland, T. Dietary quality and tree cover in Africa. Glob. Environ. Change 24, 287–294 (2014).

24. Torheim, L. E. et al. Nutrient adequacy and dietary diversity in rural Mali: association and determinants. Eur. J. Clin. Nutr. 58, 594–604 (2004).

25. Bostedt, G., Hörnell, A. & Nyberg, G. Agroforestry extension and dietary diversity – an analysis of the importance of fruit and vegetable consumption in West Pokot, Kenya. Food Secur. 8, 271–284 (2016).

26. Powell, B., Bezner Kerr, R., Young, S. L. & Johns, T. The determinants of dietary diversity and nutrition: ethnonutrition knowledge of local people in the East Usambara Mountains, Tanzania. J. Ethnobiol. Ethnomedicine 13, 23 (2017).

27. Workicho, A. et al. Household dietary diversity and Animal Source Food consumption in Ethiopia: evidence from the 2011 Welfare Monitoring Survey. BMC Public Health 16, 1192 (2016).

28. Alkire, S. & Santos, M. E. Measuring Acute Poverty in the Developing World: Robustness and Scope of the Multidimensional Poverty Index. World Dev. 59, 251–274 (2014).

29. Fatch, P. et al. Agricultural diversity linkage to income, wealth, diets and nutrition: Case of Lilongwe district in Malawi. Sci. Afr. 19, e01569 (2023).

30. Chegere, M. J. & Stage, J. Agricultural production diversity, dietary diversity and nutritional status: Panel data evidence from Tanzania. World Dev. 129, 104856 (2020).

31. Ecker, O. Agricultural transformation and food and nutrition security in Ghana: Does farm production diversity (still) matter for household dietary diversity? Food Policy 79, 271–282 (2018).

32. Jones, A. D. On-Farm Crop Species Richness Is Associated with Household Diet Diversity and Quality in Subsistence- and Market-Oriented Farming Households in Malawi. J. Nutr. 147, 86–96 (2017).

33. Sibhatu, K. T., Krishna, V. V. & Qaim, M. Production diversity and dietary diversity in smallholder farm households. Proc. Natl. Acad. Sci. 112, 10657–10662 (2015).

34. Euler, M., Krishna, V., Schwarze, S., Siregar, H. & Qaim, M. Oil Palm Adoption, Household Welfare, and Nutrition Among Smallholder Farmers in Indonesia. World Dev. 93, 219–235 (2017).

35. FAO. Guidelines on Methods for Estimating Livestock Production and Productivity. https://www.fao.org/3/ca6400en/ca6400en.pdf (2018).

36. Imai, K., Keele, L., Tingley, D. & Yamamoto, T. Causal Mediation Analysis Using R. in Advances in Social Science Research Using R (ed. Vinod, H. D.) vol. 196 129–154 (Springer New York, New York, NY, 2010).

37. Tingley, D., Yamamoto, T., Hirose, K., Keele, L. & Imai, K. mediation: R Package for Causal Mediation Analysis. J. Stat. Softw. 59, 1–38 (2014).

38. Lee, S., Lei, M. K. & Brody, G. H. Constructing Confidence Intervals for Effect Size Measures of an Indirect Effect. Multivar. Behav. Res. 50, 600–613 (2015).

39. MacKinnon, D. P., Warsi, G. & Dwyer, J. H. A Simulation Study of Mediated Effect Measures. Multivar. Behav. Res. 30, 41–62 (1995).

40. Imai, K., Keele, L. & Yamamoto, T. Identification, Inference and Sensitivity Analysis for Causal Mediation Effects. Stat. Sci. 25, (2010).

41. Cinelli, C. & Hazlett, C. Making Sense of Sensitivity: Extending Omitted Variable Bias. J. R. Stat. Soc. Ser. B Stat. Methodol. 82, 39–67 (2020).

42. Gilbert, R., Benson, T. & Ecker, O. Are Malawian Diets Changing? An Assessment of Nutrient Consumption and Dietary Patterns Using Household-Level Evidence from 2010/11 and 2016/17. https://ebrary.ifpri.org/digital/collection/p15738coll2/id/133522 (2019).

43. Jones, A. D., Shrinivas, A. & Bezner-Kerr, R. Farm production diversity is associated with greater household dietary diversity in Malawi: Findings from nationally representative data. Food Policy 46, 1–12 (2014).

44. USAID. Agriculture and Food Security | Malawi. U.S. Agency for International Development https://www.usaid.gov/malawi/agriculture-and-food-security (2023).

45. CIAT & World Bank. Climate-Smart Agriculture in Malawi. 30 https://climateknowledgeportal.worldbank.org/sites/default/files/2019-06/CSA%20_Profile_Malawi.pdf (2018).

46. Kawaye, F. P. & Hutchinson, M. F. Are Increases in Maize Production in Malawi Due to Favourable Climate or the Farm Input Subsidy Program (FISP)? in Theory and Practice of Climate Adaptation (eds. Alves, F., Leal Filho, W. & Azeiteiro, U.) 375–390 (Springer International Publishing, Cham, 2018).

47. Coulibaly, J. Y., Chiputwa, B., Nakelse, T. & Kundhlande, G. Adoption of agroforestry and the impact on household food security among farmers in Malawi. Agric. Syst. 155, 52–69 (2017).

48. Mbow, C. & Rosenzweig, C. Chapter 5: Food Security. 1–200 (2019).

49. Jamnadass, R. H. et al. Improving livelihoods and nutrition in sub-Saharan Africa through the promotion of indigenous and exotic fruit production in smallholders' agroforestry systems: a review. Int. For. Rev. 13, 338–354 (2011).

50. Ajefu, J. B. & Abiona, O. The Mitigating Impact of Land Tenure Security on Drought-Induced Food Insecurity: Evidence from Rural Malawi. J. Dev. Stud. 56, 2169–2193 (2020).

51. Peters, P. E. & Kambewa, D. Whose Security? Deepening Social Conflict Over 'Customary' Land in the Shadow of Land Tenure Reform in Malawi. J. Mod. Afr. Stud. 45, 447–472 (2007).

52. Gelli, A. et al. Value chains to improve diets: Diagnostics to support intervention design in Malawi. Glob. Food Secur. 25, 100321 (2020).

53. Forsythe, L., Nyamanda, M., Mbachi Mwangwela, A. & Bennett, B. Beliefs, Taboos and Minor Crop Value Chains. Food Cult. Soc. 18, 501–517 (2015).

54. Maliwichi-Nyirenda, C. P. & Maliwichi, L. L. Food Related Taboos Observed During Pregnancy in Malawi. Stud. Ethno-Med. 10, 263–268 (2016).

55. Beegle, K. & Poulin, M. Migration and the Transition to Adulthood in Contemporary Malawi. Ann. Am. Acad. Pol. Soc. Sci. 648, 38–51 (2013).

56. Patel, R., Bezner Kerr, R., Shumba, L. & Dakishoni, L. Cook, eat, man, woman: understanding the New Alliance for Food Security and Nutrition, nutritionism and its alternatives from Malawi. J. Peasant Stud. 42, 21–44 (2015).

57. Rasmussen, L. V. et al. Forest pattern, not just amount, influences dietary quality in five African countries. Global Food Security 25, 100331 (2020).

58. World Health Organization & FAO. Vitamin and mineral requirements in human nutrition, 2nd ed. World Health Organization. https://iris.who.int/handle/10665/42716. (2004).
