# Supplementary material for: Food-sourcing from on-farm trees mediates positive relationships between tree cover and dietary quality in Malawi
Source: Nat Food. 2024 Aug 13;5(8):661–6. doi: 10.1038/s43016-024-01028-4 (PMC11343702; doi:10.1038/s43016-024-01028-4)
Supplement: Supplementary file 2 — Reporting Summary [file 43016_2024_1028_MOESM2_ESM.pdf]

nature portfolio

Corresponding author(s): Emilie Vansant

Last updated by author(s): Jul 9, 2024

Reporting Summary

Nature Portfolio wishes to improve the reproducibility of the work that we publish. This form provides structure for consistency and transparency in reporting. For further information on Nature Portfolio policies, see our Editorial Policies and the Editorial Policy Checklist.

Statistics

For all statistical analyses, confirm that the following items are present in the figure legend, table legend, main text, or Methods section.

n/a	Confirmed	
☐	☒	The exact sample size (*n*) for each experimental group/condition, given as a discrete number and unit of measurement
☐	☒	A statement on whether measurements were taken from distinct samples or whether the same sample was measured repeatedly
☐	☒	The statistical test(s) used AND whether they are one- or two-sided<br>*Only common tests should be described solely by name; describe more complex techniques in the Methods section.*
☐	☒	A description of all covariates tested
☐	☒	A description of any assumptions or corrections, such as tests of normality and adjustment for multiple comparisons
☐	☒	A full description of the statistical parameters including central tendency (e.g. means) or other basic estimates (e.g. regression coefficient) AND variation (e.g. standard deviation) or associated estimates of uncertainty (e.g. confidence intervals)
☐	☒	For null hypothesis testing, the test statistic (e.g. *F*, *t*, *r*) with confidence intervals, effect sizes, degrees of freedom and *P* value noted<br>*Give P values as exact values whenever suitable.*
☒	☐	For Bayesian analysis, information on the choice of priors and Markov chain Monte Carlo settings
☐	☒	For hierarchical and complex designs, identification of the appropriate level for tests and full reporting of outcomes
☒	☐	Estimates of effect sizes (e.g. Cohen's *d*, Pearson's *r*), indicating how they were calculated

Our web collection on statistics for biologists contains articles on many of the points above.

Software and code

Policy information about availability of computer code

Data collection	The household surveys were designed using online using Qualtrics XM (accessed 2021-2022, no specific version available). The Qualtrics XM license agreement package included access to an app through which to conduct offline surveys. We used this to administer our survey on tablets using the Qualtrics Offline Surveys App (Version 17).
Data analysis	Survey data was downloaded to CSV format and accessed through Excel 2016 software. The CSV file was uploaded to ArcGIS Pro (Version 3.0.3) to displace and map household GPS points.<br><br>For each respondent's food consumption data, we also generated Estimated Usual Intake values using the Multiple Source Method (MSM). This method synthesizes the multiple dietary surveys per respondent to adjust for interpersonal variation in consumption patterns. We used a web-based statistics package developed by the German Institute of Human Nutrition (DIFE) to implement the MSM with our nutrition data: https://nugo.dife.de/msm.<br><br>To examine the relationship between tree cover, use of on-farm food trees, and dietary quality, we cleaned and analyzed our data in R (version 4.2.2). We ran linear regressions, controlling for a selection of covariates, to examine these relationships. Specifically, we used the 'mediation' package in R to evaluate the causal mediation (i.e. indirect) effect of our food tree variable. This package also allowed us to evaluate the robustness of our average causal mediation effect estimates by testing for the possible confounding effect of unmeasured pretreatment variables. We also tested for the impact of omitted variables using the 'sensmakr' package in R.

For manuscripts utilizing custom algorithms or software that are central to the research but not yet described in published literature, software must be made available to editors and reviewers. We strongly encourage code deposition in a community repository (e.g. GitHub). See the Nature Portfolio guidelines for submitting code & software for further information.

Data

Policy information about availability of data

All manuscripts must include a data availability statement. This statement should provide the following information, where applicable:

- Accession codes, unique identifiers, or web links for publicly available datasets
- A description of any restrictions on data availability
- For clinical datasets or third party data, please ensure that the statement adheres to our policy

The dataset generated by the survey research and the code to facilitate the replication of this study is publicly available at: https://doi.org/10.7910/DVN/WBUTCK
We also accessed very high resolution (3m) tree cover data from 2019 PlanetScope nanosatellite constellation imagery: https://www.nature.com/articles/s41467-023-37880-4
Estimated nutrient intakes were calculated using the following publicly available Food Composition Tables:
Malawi: http://hdl.handle.net/10427/D217R336D
Tanzania: https://nutritionsource.hsph.harvard.edu/food-tables/
Zambia: https://nfnc.org.zm/download/zambia-food-composition-tables-4th-edition/
Mozambique: http://hdl.handle.net/10138/337295
Kenya: https://nutritionhealth.or.ke/programmes/healthy-diets-physical/food-composition-tables/
West Africa: https://openknowledge.fao.org/server/api/core/bitstreams/c5b37ac2-7082-48ab-a4a5-68d27deb4849/content
United States (USDA): https://fdc.nal.usda.gov/

Research involving human participants, their data, or biological material

Policy information about studies with human participants or human data. See also policy information about sex, gender (identity/presentation), and sexual orientation and race, ethnicity and racism.

Reporting on sex and gender	For our household and dietary surveys, we targeted women in each household with children between the ages of 2-5 years. The focus was on gender, rather than biological sex, due to the cultural and societal role women play in Malawian households in determining patterns of food consumption. Consent was obtained for sharing individual-level data. As all of our individual data was collected from people who self-identified as women, no gender-based analysis was performed.
Reporting on race, ethnicity, or other socially relevant groupings	In the survey, we asked respondents to self-report their ethnicity (the question was phrased as "What is your ethnic group?" and translated accordingly). No data was collected on race or any other social grouping. Reporting on ethnicity was not included in the data analysis or manuscript. This is because the village areas selected in each study region were ethnically homogenous, with the village area populations predominantly (>90%) identifying as either Tonga/Tumbuka (Nkhata Bay) or Lomwe (Mulanje). It is noted that in terms of food culture, Tonga and Tumbuka cultures are regarded as similar based on mutual linguistic intelligibility and shared cultural history relative to other ethnic groups in Malawi (Kamwendo, 2005). Therefore, given the strong correlation between ethnicity and study region, we decided to only use the latter variable in our analysis.<br><br>Kamwendo, G. (2005). Language, Identity and the Politics of Recognition in Post-Banda Northern Malawi. Ufahamu: A Journal of African Studies, 31(1–2). https://doi.org/10.5070/F7311-2016524
Population characteristics	The human research participants were women, aged 18-47 (average: 28). At the time of the dry season survey, 38 women were pregnant, 143 reported to be breastfeeding. At the time of the wet season survey, 30 women were pregnant, 119 reported to be breastfeeding. We selected women with at least one child between the ages of 2 – 5 years as the primary survey respondents. This was to target women of reproductive age and focus on women feeding their children solid foods. In Malawi, women play an integral role in household food security and nutrition. They are traditionally responsible for food selection, preparation, and feeding of dependents (elders/children). Women's dietary diversity has been shown to align with household dietary diversity, which indicates that women are reliable representatives of dietary quality at the household level.<br><br>No other data was collected regarding respondent health status or other physical conditions.
Recruitment	Prior to recruitment, local authorities in each village area were visited to obtain permission to conduct research activities in each community. Individual survey participants were then recruited by local Health Surveillance Assistants (HSAs), who had records on women in each village area corresponding to our recruitment criteria (women with children between the ages of 2-5 years). After obtaining a full list of eligible women in each village area, we conducted systematic sampling (choosing a random starting point and then selecting every nth woman on the list relative to list length/village size). The list of women was not in any particular order, so we can assume there was no hidden pattern that would skew the sample. In the event that a recruited respondent was not available for the survey, the following household on the HSA's list was selected. Consent forms in the local language were read and signed by recruited survey participants to ensure voluntary participation and optional withdrawal at any time in the study prior to data collection.
Ethics oversight	The project has received approval from the European Research Council (funding the parent project under the European Union's Horizon 2020 research and innovation programme) and the Research Ethics Committee for Science and Health at the University of Copenhagen (hosting institution).

Note that full information on the approval of the study protocol must also be provided in the manuscript.

Field-specific reporting

Please select the one below that is the best fit for your research. If you are not sure, read the appropriate sections before making your selection.

☐ Life sciences ☒ Behavioural & social sciences ☐ Ecological, evolutionary & environmental sciences

For a reference copy of the document with all sections, see nature.com/documents/nr-reporting-summary-flat.pdf

Behavioural & social sciences study design

All studies must disclose on these points even when the disclosure is negative.

Study description

We analyzed the relationship between tree cover, household decisions to source food from on-farm trees and women's micronutrient adequacy in rural Malawi. As part of a mixed-methods case study, we collected quantitative and qualitative data in the dry season (October 2021) and wet season (March 2022). Our analysis includes data from 460 women with whom we conducted surveys in both seasons, including 24-hr dietary recall data on food consumption.

Research sample

Our survey was designed to collect data at the individual and household level. At the individual level, our research sample consisted of women (aged 18 - 48; average 28). Given the age range and average, we find this sample to be adequately representative of women of reproductive age in Malawi - our target demographic for the survey. Our sample consisted of women with at least one child between the ages of 2 – 5 years as the primary survey respondents. This was to target women of reproductive age and focus on women feeding their children solid foods. In Malawi, women play an integral role in household food security and nutrition. They are traditionally responsible for food selection, preparation, and feeding of dependents (elders/children). Women's dietary diversity has been shown to align with household dietary diversity, which indicates that women are reliable representatives of dietary quality at the household level.

Sampling strategy

Across the two districts, we initially surveyed 515 women (dry season) and followed-up with 460 women (wet season) (89% attrition rate). No statistical methods were used to predetermine sample size. The sample size for the dry season was set at 500, with 15 extra households added due to available time and resources. Upon consulting experts in this field of nutrition, this sample size was determined as sufficient for a study using a 24-hour dietary recall (a time-intensive survey tool). The sample size for the wet season was determined by the availability of respondents - as we had to track down all respondents we had previously visited for the first round of the survey.

Data collection

The household and 24-hr dietary recall surveys were tested and validated by a team of six MSc Nutrition graduates from the Lilongwe University of Agriculture and Natural Resources (LUANAR), who were hired as enumerators to conduct the surveys in the local language, Chichewa. All enumerators also contributed to the translation and revision of the household and dietary data survey tools. Therefore no researchers were blind to the study design nor research hypotheses during data collection. Household surveys were administered using tablets (Qualtrics XM app) with questions pertaining to household characteristics, household assets, farming systems, forest use, and respondents' food consumption. We used a quantitative, 24-hr dietary recall survey to collect detailed information on the type, quantity, and source of the foods people consumed the previous day. Photo aids and local serving size aids (plates, bowls, cups) were used to help respondents estimate the quantities of food and drink items consumed.

Enumerators were trained to interview participants in isolation to avoid biases, with the exception of minors under the care of the respondent. Multiple 24-hr recalls at different times of the year have shown to be useful in accounting for seasonal variation in food intake, and multiple day 24-hr recalls with the same individual are integral to capturing variability in food intake. To reduce systematic error and bias in the dietary data collection, an interview protocol with culturally sensitive tools and methods was developed in close collaboration with local enumerators. For both rounds of fieldwork, each household was visited on two non-consecutive days within a 7-day period. The first visit consisted of conducting a combined household/dietary recall survey, and the second visit consisted of a follow-up dietary recall. Conducting two dietary recalls per respondent allowed us to obtain a more accurate 'snapshot' of habitual food consumption for each respondent, thereby improving our ability to calculate their estimated usual intakes for each target nutrient in each season (see Data Analysis section).

Timing

The first round of data collection was conducted from 20/10/2021 - 24/11/2021, and the second round was conducted from 01/03/2022 - 28/03/2022.

Data exclusions

In our analysis, we excluded the 55 households for which we only had household and dietary data from the dry season survey.

Non-participation

No participants declined participation in the survey. In the follow-up (wet season) round of data collection, we were unable to re-visit 55 households. This is because the woman who we had originally surveyed was unavailable for reasons including: seasonal migration, family illness, funeral attendance. When possible, all efforts were made to follow-up with individual respondents within the survey time-frame.

Randomization

Study sites (ie. village clusters) were intentionally chosen in each district based on proximity to forests and markets. Eligible respondents corresponding to our pre-determined study criteria were randomly selected from lists provided by local health authorities (taking every nth household on the list, depending on village size). Post data collection, we compared women in areas with different degrees of tree cover and controlled for potential confounding variables that could explain differences in food consumption, including: household size, the Multidimensional Poverty Index (MPI) living standard, farm size (acres under cultivation), education level, livestock holdings (TLU), crop count, and study region. This selection of covariates was informed by a synthesis of studies linking trees and dietary quality as well as extensive fieldwork in Malawi.

Reporting for specific materials, systems and methods

We require information from authors about some types of materials, experimental systems and methods used in many studies. Here, indicate whether each material, system or method listed is relevant to your study. If you are not sure if a list item applies to your research, read the appropriate section before selecting a response.

Materials & experimental systems

n/a	Involved in the study
☒	☐ Antibodies
☒	☐ Eukaryotic cell lines
☒	☐ Palaeontology and archaeology
☒	☐ Animals and other organisms
☒	☐ Clinical data
☒	☐ Dual use research of concern
☒	☐ Plants

Methods

n/a	Involved in the study
☒	☐ ChIP-seq
☒	☐ Flow cytometry
☒	☐ MRI-based neuroimaging

Plants

Seed stocks	*Report on the source of all seed stocks or other plant material used. If applicable, state the seed stock centre and catalogue number. If plant specimens were collected from the field, describe the collection location, date and sampling procedures.*
Novel plant genotypes	*Describe the methods by which all novel plant genotypes were produced. This includes those generated by transgenic approaches, gene editing, chemical/radiation-based mutagenesis and hybridization. For transgenic lines, describe the transformation method, the number of independent lines analyzed and the generation upon which experiments were performed. For gene-edited lines, describe the editor used, the endogenous sequence targeted for editing, the targeting guide RNA sequence (if applicable) and how the editor was applied.*
Authentication	*Describe any authentication procedures for each seed stock used or novel genotype generated. Describe any experiments used to assess the effect of a mutation and, where applicable, how potential secondary effects (e.g. second site T-DNA insertions, mosiacism, off-target gene editing) were examined.*
